# Supplementary material for: Chromothripsis during telomere crisis is independent of NHEJ, and consistent with a replicative origin
Source: Genome Res. 2019 May;29(5):737–49. doi: 10.1101/gr.240705.118 (PMC6499312; doi:10.1101/gr.240705.118)
Supplement: Supplemental Material [file supp_gr.240705.118_Supplemental_file_1.zip › contigs/annotated_contigs/DB82/contig.2.DB82_length_377_mean_cov_7.68169761273.docx]

**DB82_length_377_mean_cov_7.68169761273**

```
TTTCTTTTTCCTTCTTTCTTTCTTTTTCTTTCTTTCTTTTTCCTTCTTTCTTTCTTTTTCTTTTTCTTTCTTCTTTCTGTCTTTCTTTC
 >chr11:39199151-39199424 - E=5e-150
CTTTCTCTCCCTCTCTCCCTCTCTTCCTCTTCCCCTTCCCTTTCCCTTTCTTTCCTGATTAAACATAAGACATTTATTTCTCACAGTTC

TGGAAGCTGGGAAGTCCAAAATCAAGGTGCAGGTAGATCTGTTGTCTGGTGAGGGCATTCTTTTTGGCTTGAAGATAGATGTCTTTTTG

TTGG|AT|ATATATATATATATACACCTGAAGGATTATAAATCATTCTACCATAAAGACACATACACATGTGTGTTTATTGTAGCACTA
     >chr11:39173339-39173445 - E=2e-52
TTCACAATAGCAAAGACTTGGAA
```
